# Preventive Effect of Dihydromyricetin against Cisplatin-Induced Nephrotoxicity* In Vitro* and* In Vivo*

**DOI:** 10.1155/2016/7937385

**Published:** 2016-08-24

**Authors:** Fei Wu, Yi Li, Haibo Song, Yuan Zhang, Yuzhu Zhang, Miao Jiang, Fang Wang, Qian Mu, Wen Zhang, Liang Li, Dongqi Tang

**Affiliations:** ^1^Center for Stem Cell & Regenerative Medicine, The Second Hospital of Shandong University, Jinan 250012, China; ^2^Department of Joint Surgery, Shandong Provincial Hospital Affiliated to Shandong University, Jinan, Shandong 250021, China

## Abstract

Nephrotoxicity is a frequent severe side effect of cisplatin chemotherapy, limiting its clinical use despite being one of the most potent chemotherapy drugs. Dihydromyricetin is a highly abundant compound purified from the leaves of* Ampelopsis grossedentata*. Previous studies have demonstrated the anti-inflammatory and antioxidative effects of Dihydromyricetin both* in vitro* and* in vivo*, but little is known about the effects of Dihydromyricetin on cisplatin-induced nephrotoxicity and its underlying mechanisms. In the present study, we investigated its potential renoprotective effect and found that Dihydromyricetin ameliorated the renal functional impairment and structural damage caused by cisplatin. Moreover, Dihydromyricetin markedly attenuated cisplatin-induced oxidative stress, as well as protecting against cisplatin-induced inflammation and apoptotic cell death in mouse kidney tissues. These results collectively highlight the potential of DMY as a rational renoprotective agent against cisplatin.

## 1. Introduction

Cisplatin, which belongs to platinum-based antineoplastic drugs, is one of the major antineoplastic and highly effective drugs which has been demonstrated to have one of the highest cure rates [1]. Cisplatin, in combination with related platinum-based therapeutics, has been used for the treatment of bladder, testicular, ovarian, cervical, head and neck, small-cell and non-small cell lung carcinoma, and many other types of cancers [2–4]. However, the severe side effects in normal tissues, especially in kidneys, greatly limit its chemotherapeutic efficacy [1, 5]. It has been reported that approximately one-third of patients receiving high-dose cisplatin treatment experience cisplatin nephrotoxicity leading to a subclinical but permanent reduction of renal function [3, 6]. For decades, numerous studies have focused on investigating the mechanisms of nephrotoxicity development, and it has been demonstrated that inflammation, oxidative stress injury, mitochondrial dysfunction, and direct cytotoxicity to the tubular epithelial cells may partially explain this process [3, 7].

Dihydromyricetin (DMY), also known as ampelopsin, is a natural flavonoid compound extracted from the leaves of ampelopsis grossedentata, which is commonly known as vine tea. Vine tea is considered to have numerous pharmacological functions, is used in traditional Chinese medicine to accelerate detoxification of ethanol and treat fever and cough, and possesses antioxidative and antimicrobial properties [8, 9]. As the most abundant and important bioactive ingredient in vine tea, DMY has been reported to exert anti-inflammatory, antioxidant, and anticancer effects in previous research [10–12]. Zhu et al. have demonstrated that DMY exerts a cardioprotective effect against adriamycin-induced injury in ICR mice [13]. However, little is known about the effect of DMY in cisplatin-induced nephrotoxicity.

In the current study, we investigated the renoprotective effect of DMY in a cisplatin-treated mouse model by measuring the renal functional parameters of serum creatinine (SCr) and blood urea nitrogen (BUN) levels and histological examination using periodic acid-Schiff (PAS) staining. We found that DMY decreased the malondialdehyde (MDA) level and increased the catalase activity (CAT) and superoxide dismutase (SOD) activities in mouse kidney tissues after treatment with cisplatin for 3 days. We also observed decreased mRNA expression of several proinflammatory cytokines. Moreover, DMY mediated a protective effect against cisplatin-induced nephrotoxicity by ameliorating apoptotic cell death in HK-2 cells.

## 2. Materials and Methods

### 2.1. Animals

Adult male C57BL/6J mice (6–8 weeks old, 20–25 g body weight) were used in this study. These mice were purchased from Shanghai SLAC Laboratory Animal Co., Ltd., and housed at 22 ± 2°C and 60 ± 5% humidity on a 10–12 h light-dark cycle under a pathogen-free condition. All animals had free access to sterile tap water and food during the experiments. All of the animal experimental protocols were performed in accordance with the NIH Guide for Care and Use of Laboratory Animals and were approved by the Institutional Animal Care and Use Committee at Shandong University, China.

### 2.2. Cisplatin-Induced Nephrotoxicity

DMY (Aladdin) was suspended in sesame oil and administrated by gavage to C57BL/6J mice. Cisplatin (Sigma-Aldrich) was dissolved in 0.9% saline at a concentration of 2 mg/mL and mice were given a single intraperitoneal injection. The mice were randomly divided into a control group, 30 mg/kg cisplatin-treated group, 30 mg/kg cisplatin and 100 mg/kg DMY-treated group, 30 mg/kg cisplatin and 300 mg/kg DMY-treated group, and 30 mg/kg cisplatin and 500 mg/kg DMY-treated group. The DMY treatment was started 4 days prior to a single i.p. injection of cisplatin and continued for an additional 3 days.

### 2.3. Sample Collection

Mice were anesthetized and sacrificed 3 days after cisplatin injection. Blood samples were collected through tail vein and serum was separated and used for the assessment of serum creatinine concentration (SCr) and blood urea nitrogen (BUN). Next, hearts were cannulated with 0.9% NaCl at 90 mmHg via the left ventricular apex to clear the kidneys until being whitish. Both kidneys were removed and stored in liquid nitrogen or fixed in 4% paraformaldehyde and embedded in paraffin for histological analysis.

### 2.4. Assessment of Renal Function

Blood samples were transferred to 1.5 mL tubes and allowed to clot. Then, serum was separated by centrifugation at 4000 rpm for 15 min. SCr and BUN were measured using a Beckman Coulter Dxc800 biochemical analyzer (Beckman Coulter).

### 2.5. Evaluation of Histology and Immunohistochemistry

Formalin-fixed and paraffin-embedded kidney tissues were sectioned at 4 *μ*m and stained with periodic acid-Schiff (PAS) for histological examination. Apoptotic cells were detected using the TdT-mediated dUTP nick-end labeling (TUNEL) assay using an* in situ* apoptosis detection kit (Roche Applied Science) according to the manufacturer's protocol. Immunohistochemical staining for 8-hydroxydeoxyguanosine (8-OHdG) was performed using anti-8-OHdG antibody (Santa Cruz Biotechnology). In the evaluation of hematoxylin and eosin (HE) stained sections, interstitial polymorphonuclear leukocyte (PMN) infiltration was scored by a renal pathologist who was blinded to the experimental groups. PMN infiltration per high power (×200) field was graded semiquantitatively according to the following schema: 0, 0-1; 1, 2–10; 2, 11–20; 3, 21–40; 4, >40 or too many to count [14].

### 2.6. Measurement of Oxidative Stress Markers

Oxidative stress markers were measured in kidney tissues of the mice. Kidney tissues were homogenized in lysis buffer and the supernatant was used for the measurement of malondialdehyde (MDA), superoxide dismutase (SOD), and catalase activity (CAT).

Determination of kidney tissue MDA concentration was measured using a Lipid Peroxidation MDA Assay kit (Beyotime Biotechnology). The concentration of MDA was detected by reacting with thiobarbituric acid (TBA). Briefly, 0.1 mL of the kidney tissue supernatant was mixed with 200 *μ*L MDA working solution, heated in a heat block (100°C) for 15 min, and then cooled down to room temperature. After centrifugation at 1000 g for 10 min, the supernatant was measured at a wavelength of 532 nm.

The assay for SOD activity was based on the ability of SOD to inhibit nitroblue tetrazolium (NBT) reduction by superoxide. Briefly, 20 *μ*L of the tissue supernatant was mixed with 160 *μ*L NBT working solution and 20 *μ*L reacting solution and then incubated in 37°C for 30 min. Finally, the optical density was measured at a wavelength of 560 nm.

The catalase activity was detected using a Catalase Analysis Kit (Beyotime Biotechnology) according to the manufacturer's instructions. Briefly, the tissue supernatant was treated with excess hydrogen peroxide for decomposition for an indicated time. The remaining hydrogen peroxide coupled with a substrate was treated with peroxidase to produce N-4-antipyryl-3-chloro-5-sulfonate-p-benzoquinonemonoimine, which absorbs maximally at a wavelength of 520 nm.

### 2.7. Cell Culture

HK-2 human kidney proximal tubular cells were purchased from American Type Culture Collection and cultured in renal epithelial basal medium (Gibco) with manufacturer provided supplements in an incubator with 5% CO_2_ at 37°C.

### 2.8. Cell Viability Assay

The cytotoxicity of cisplatin was determined by the water-soluble tetrazolium- (WST-) 1 method using the WST-1 cell proliferation and cytotoxicity assay kit (Beyotime Biotechnology). Briefly, HK-2 were seeded in 96-well plates at 5000 cells/well and incubated at 37°C for 24 h. The media were replaced with normal media in the absence or presence of DMY (25 or 50 *μ*M). After incubation for 24 h, cells were exposed to cisplatin (10, 20, 30, or 50 *μ*M) for another 24 h. Then WST-1 reagent was added for 4 h at 37°C, and absorbance was measured at 450 nm wavelength using an automated microplate reader.

### 2.9. DAPI Staining Assay

HK-2 cells were pretreated with or without DMY for 24 h and then treated with or without cisplatin for another 24 h. Cells were washed twice with PBS and then fixed with 4% paraformaldehyde and permeabilized with 0.5% triton X-100 for 10 min. After that, cells were incubated in 4′,6-diamidino-2-phenylindole (DAPI)/PBS (1 : 5000 dilution, Sigma) for 3 min at room temperature. Finally, the nuclei were photographed using fluorescence microscopy.

### 2.10. Western Blot Analysis

Equal amounts of total protein were separated by sodium dodecyl sulfate polyacrylamide gels (SDS-PAGE) and subsequently transferred to polyvinylidene fluoride (PVDF) membranes. After blocking in 5% nonfat dry milk for 1 h, membranes were incubated with primary antibodies against Bcl-2 (1 : 1000 dilution), Bax (1 : 1000 dilution), and GAPDH (1 : 1000 dilution) overnight at 4°C. Membranes were washed three times in 0.01 M Tris Buffered Saline with 0.1% Tween-20 (TBST) and then incubated in HRP-labeled secondary antibody (1 : 5000 dilution, anti-mouse) for 1 h and detected using enhanced chemiluminescence (ECL) solution.

### 2.11. Quantitative Real-Time-Polymerase Chain Reaction (Q-PCR)

For Q-PCR analysis, total RNA was extracted using the TRIzol reagent (Invitrogen). cDNA was prepared using Revert Aid First Strand cDNA Synthesis kit (Thermo Scientific) according to the manufacturer's protocol. Real-time PCR in triplicate was performed with SYBR Green Master Mix (Applied Biosystems). A standard amplification protocol was used for real-time PCR analysis: 95°C for 10 s, followed by 40 cycles of 95°C for 5 s, 60°C for 30 s, and a final extension at 72°C for 3 min. Primers that were used for Q-PCR are summarized in Table 1.

### 2.12. Statistical Analysis

Data are presented as mean ± SD. Statistical analysis was conducted using the SPSS software. All experiments were repeated at least three times to obtain the data. Statistical evaluation among multiple groups was performed by one-way ANOVA followed by Student's* t*-test for analysis of differences in each group. A* P* value <0.05 was considered as statistically significant.

## 3. Results

### 3.1. Effect of Dihydromyricetin on Cisplatin-Induced General Toxicity and Nephrotoxicity in Mice

**Figure 1:**
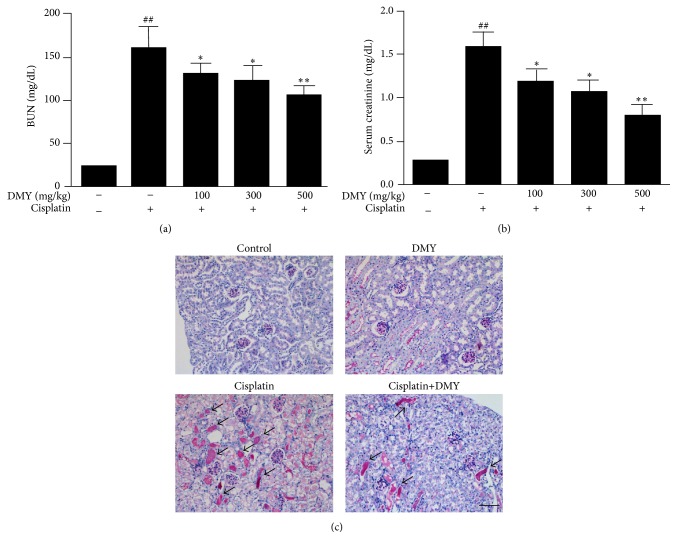
Dihydromyricetin protected against cisplatin-induced general toxicity and nephrotoxicity* in vivo*. (a and b) BUN and SCr measurement. (c) Periodic acid-Schiff (PAS) staining. Kidney sections from the cisplatin-treated group showed severe cast formation and severe tubular injury. Cisplatin with DMY (500 mg/kg) showed less tubular destruction and mild casts (indicated with arrows). Data are presented as mean ± SD. ^*∗∗*^ *P* < 0.01 versus cisplatin treatment group, ^*∗*^ *P* < 0.05 versus cisplatin treatment group, and ^##^ *P* < 0.01 versus nondrug treatment group.

In order to evaluate whether pretreatment of DMY could ameliorate renal functional impairment induced by cisplatin, serum creatinine (SCr) and blood urea nitrogen (BUN) levels were measured as a marker of renal function in mice as described in Section 2. As shown in Figures 1(a) and 1(b), the cisplatin-treated group exhibited a significant increase in SCr and BUN compared with the control group. The enhanced levels of SCr and BUN were significantly decreased in the cisplatin with DMY groups in a dose-dependent manner. The DMY concentration of 500 mg/kg showed a greater protective effect and was used for all following experiments.

Furthermore, periodic acid-Schiff (PAS) staining was performed for histological examination (Figure 1(c)). As expected, the cisplatin-treated group showed severe loss of brush border and cast formation. On the contrary, DMY treatment significantly attenuated these pathological changes induced by cisplatin.

**Figure 2:**
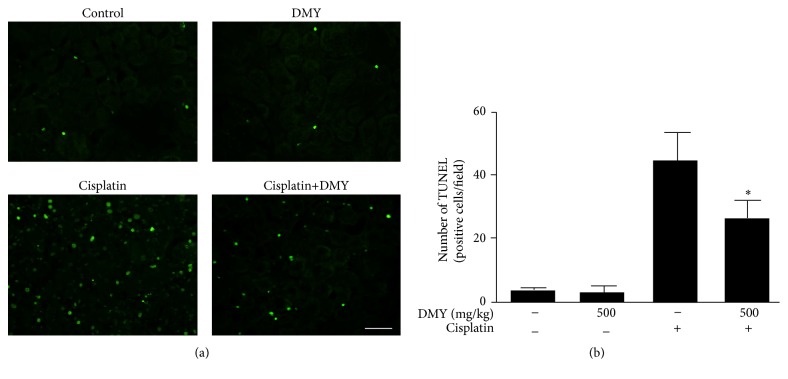
TUNEL-positive: that is, apoptotic nuclei were stained with green spot. (a) TUNEL staining demonstrated cisplatin-induced cell apoptosis in the nondrug treatment group, DMY treatment group, cisplatin treatment group, and cisplatin with DMY (500 mg/kg) treatment group. Scale bar, 50 *μ*m. (b) The TUNEL-positive cells were quantified using Imagine J software at 200x magnification. Data are presented as mean ± SD. ^*∗*^ *P* < 0.05 versus cisplatin treatment group.

Using TUNEL staining, cisplatin-administered mice also exhibited a significantly higher number of positive nuclei compared with control, while the DMY-treated group indicated a decrease in apoptotic cells (Figure 2).

### 3.2. Effect of Dihydromyricetin on Cisplatin-Induced Cell Viability and Apoptosis in HK-2 Cells

**Figure 3:**
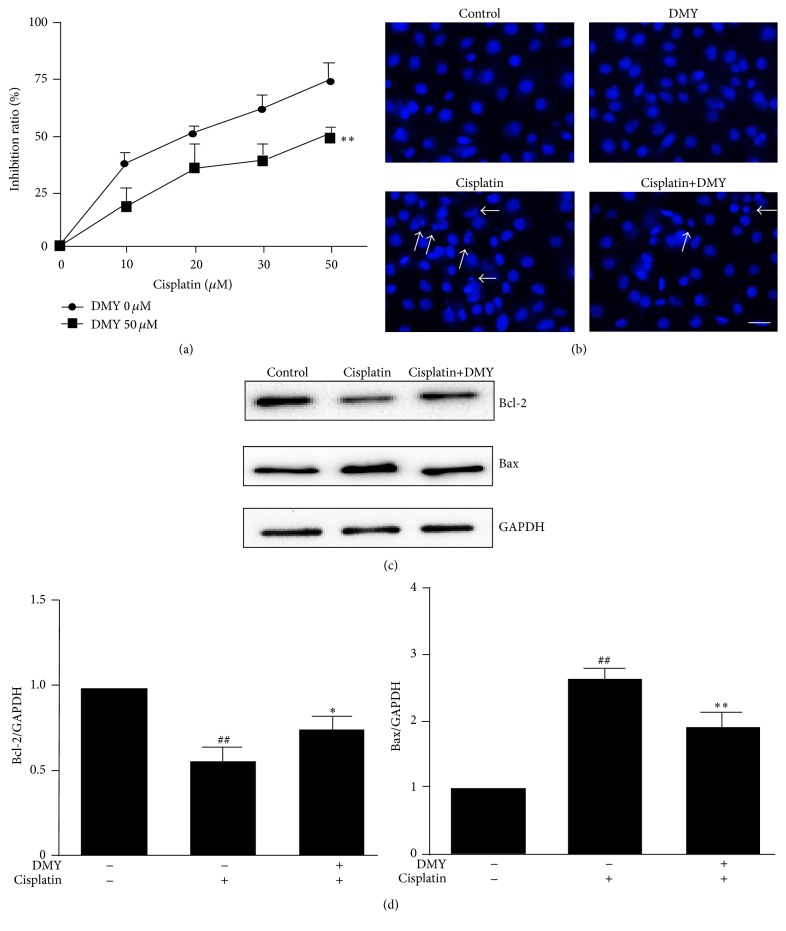
Dihydromyricetin reduced cell apoptosis caused by cisplatin in HK-2 cells. Inhibition rate (a) and cell apoptosis (b) were determined by WST-1 and DAPI staining (indicated with arrows, scale bar, 50 *μ*m) after HK-2 cells were treated with DMY (pretreated 24 h earlier than cisplatin) and cisplatin. (c) HK-2 cells were pretreated with DMY (50 *μ*M) for 24 h followed by cisplatin treatment for an additional 24 h; then the Bcl-2, Bax, and GAPDH protein expression was analyzed by Western Blot. (d) Protein bands were quantitated by densitometric analysis. Data are presented as the mean ± SD. ^*∗∗*^ *P* < 0.01 versus cisplatin treatment group, ^*∗*^ *P* < 0.05 versus cisplatin treatment group, and ^##^ *P* < 0.01 versus nondrug treatment group.

Since it is commonly recognized that nephrotoxicity caused by cisplatin is attributed to cell injury and death in renal tubules [15–17], we next examined the effect of DMY on HK-2 cell apoptosis induced by cisplatin. As shown in Figure 3(a), cell viability was inhibited by cisplatin in a concentration-dependent manner (10, 20, 30, or 50 *μ*M), while DMY effectively abated the inhibitory effects caused by cisplatin. To further validate that DMY significantly attenuated cisplatin-induced cell apoptosis in HK-2, DAPI staining was performed in the following experiment. The results demonstrated that extensive nuclear fragmentation was clearly observed in the cisplatin-treated group. On the contrary, after pretreatment with DMY for 24 h, these nuclear changes were decreased (Figure 3(b)). To further validate the impact of DMY on cisplatin-induced apoptosis, we measured the expression of the proapoptotic factor Bax and the antiapoptotic factor Bcl-2. Western blot analysis indicated that the decreased expression of Bcl-2 caused by cisplatin was partially upregulated by the pretreatment of DMY and Bax expression was downregulated in the DMY coadministration group (Figures 3(c) and 3(d)).

### 3.3. Effect of Dihydromyricetin on Oxidative Stress in Kidney Tissues

**Figure 4:**
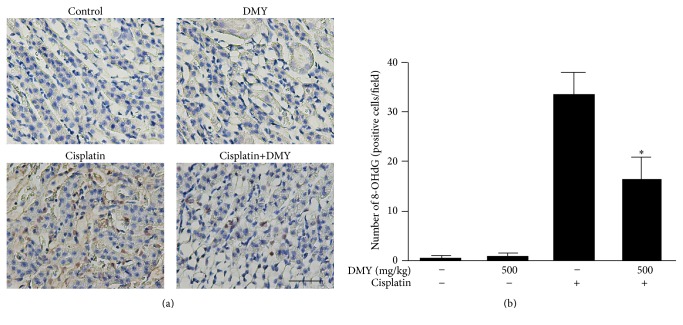
Dihydromyricetin attenuates cisplatin-induced oxidative stress. (a) Immunohistochemical staining was performed for 8-OHdG. Scale bar, 50 *μ*m. 8-OHdG-positive nuclei are stained with dense brown spots. (b) The 8-OHdG-positive cells were quantified using Imagine J software at 400x magnification. Data are presented as mean ± SD. ^*∗*^ *P* < 0.05 versus cisplatin treatment group.

For many years, oxidative stress has been recognized as one of the central players in the pathophysiology of cisplatin-induced acute kidney injury (AKI) [15, 18]. To investigate the protective mechanism of DMY on cisplatin nephrotoxicity, we next measured the level of several oxidative stress markers in nephridium. Three days after cisplatin treatment, a significant increase in tissue MDA level and decrease of CAT and SOD activities occurred compared with the control group, while pretreatment of DMY partially blocked the increase of MDA level and attenuated the reduction of these antioxidant levels (Table 2). Moreover, we also assessed 8-OHdG, a commonly used marker for the evaluation of DNA oxidization, to confirm the free radical-induced oxidative lesions in each group. As shown in Figure 4, the numbers of 8-OHdG-positive cells were much greater in the cisplatin-treated group when compared to the control mice. Also, a significant decrease of the positive cell numbers was observed in the cisplatin with Dihydromyricetin group.

### 3.4. Effect of Dihydromyricetin on Proinflammatory Mediators in Kidney Tissues

**Figure 5:**
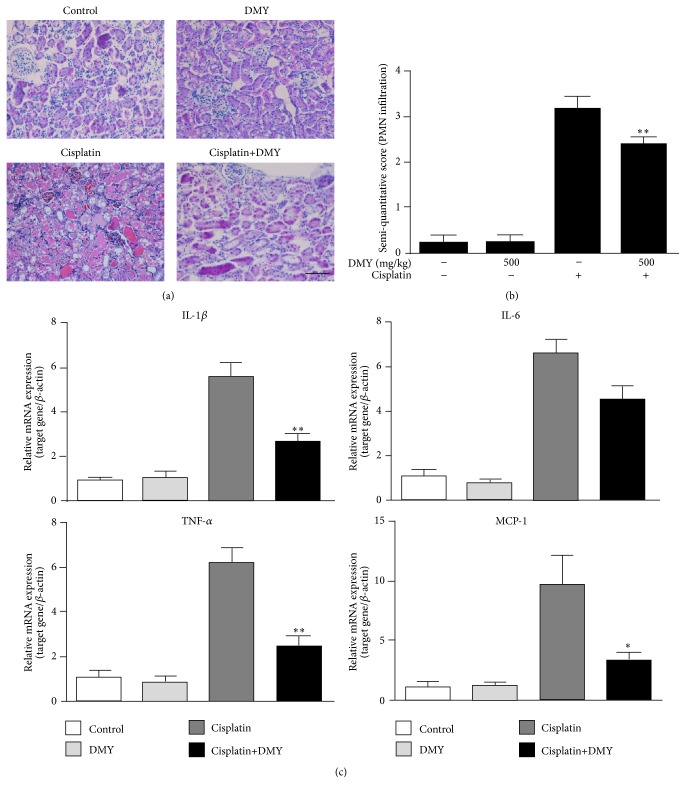
Dihydromyricetin decreased PMN infiltration and expression of several proinflammatory cytokines. (a) Hematoxylin and eosin (HE) staining was performed for the analysis of PMN infiltration. Scale bar, 50 *μ*m. (b) The semiquantitative score of PMN infiltration was measured according to Section 2. (c) q-PCR analysis of IL-1*β*, IL-6, TNF-*α*, and MCP-1 mRNA expression in the nondrug treatment group, DMY treatment group, cisplatin treatment group, and cisplatin with DMY (500 mg/kg) treatment group (*n* = 5). Data are presented as mean ± SD. ^*∗∗*^ *P* < 0.01 versus cisplatin treatment group, ^*∗*^ *P* < 0.05 versus cisplatin treatment group.

Although many studies indicate that oxidative stress plays critical roles in the pathogenesis of cisplatin-induced development of kidney damage [14, 19], AKI caused by cisplatin is also associated with a severe inflammatory response [20, 21]. To measure the potential inflammatory mechanisms, we measured the semiquantitative score of PMN infiltration by HE staining. The cisplatin-treated group had greater PMN infiltration (score: 3.25 ± 0.25 versus 2.50 ± 0.19) when compared with the DMY coadministration group (Figures 5(a) and 5(b)). Furthermore, mRNA expression of several proinflammatory cytokines was also examined (Figure 5(c)). The quantitative real-time PCR results showed that gene expression of IL-1*β*, IL-6, TNF-*α*, and MCP-1 was significantly higher after cisplatin administration, and this effect was partially mitigated by the pretreatment with DMY; however, the lower gene expression levels of IL-6 showed no significant differences when compared with the cisplatin group.

## 4. Discussion

As one of the major antineoplastic drugs for the treatment of solid tumors, cisplatin has been used for various types of cancers since it was approved for the treatment of testicular and ovarian cancers in 1978. However, in the past 30 years, the application of cisplatin has been seriously limited due to its serious side effect of dose-dependent renal toxicity [22]. Nearly 20% of the patients who received high-dose cisplatin treatment developed severe renal insufficiency. Until now, there was no specific treatment for cisplatin-induced renal dysfunction or injury. It is generally believed that cisplatin binding to DNA can not only cause cytotoxicity of tumors but also damage other proliferating cells, and nephrotoxicity primarily occurs in proximal tubule epithelial cells.

As a traditional Chinese medicine, DMY is the primary natural product extracted from ampelopsis grossedentata and has long been credited with scavenging free radical, protecting liver, and preventing some cancers. In the current study, we explored the antioxidation effect of DMY on cisplatin-induced renal toxicity and found that it can perform protective roles both* in vitro* and* in vivo*. Using the mouse model of cisplatin-induced kidney lesion, DMY could effectively ameliorate the renal functional impairment and structural damage as well as improving the animals' survival rate. In addition, it mediated a protective effect against cisplatin-induced cell apoptosis in HK-2 cells and upregulated antiapoptotic Bcl-2 levels together with downregulated proapoptotic Bax levels. Moreover, we found that DMY decreased the MDA level and increased the CAT and SOD activities in mouse kidney tissues after cisplatin treatment and also decreased the gene expression of several proinflammatory cytokines which included IL-1*β*, IL-6, TNF-*α*, and MCP-1. To the best of our knowledge, the effect of DMY on preventing cisplatin-induced nephrotoxicity might have potential clinical application for cancer treatment.

For over a decade, oxidative stress has been regarded as one of the most widely accepted factors that contribute to cisplatin nephrotoxicity. Cisplatin treatment can lead to accumulation of endogenous ROS and oxidative stress within the renal tubular cells and kidney slices, as well as* in vivo* in whole animals. Meanwhile, during cisplatin treatment, a robust inflammatory reaction occurs and inflammasomes are also stimulated, further exacerbating renal tissue damage. In our present study, we found that the pretreatment of DMY significantly attenuated cisplatin-induced nephrotoxicity not only by its antioxidation effect, but also by decreasing the expressions of several proinflammatory cytokines.

Moreover, since the key histopathological features in cisplatin nephrotoxicity are renal tubular cell injury and death, the apoptosis of HK-2 cells was also investigated in our study. The Bax and Bcl-2 proteins belong to the Bcl-2 family, which regulate and execute many cellular intrinsic apoptotic pathways and are the best-characterized group of apoptosis-regulating factors [23]. Bcl-2 has been regarded to function as an inhibitor of apoptosis, while Bax is associated with apoptotic cell death process. We found that the treatment with DMY decreased cisplatin-induced apoptosis and necrosis of HK-2 cells. The underlying mechanism was associated with the effect of DMY upregulating Bcl-2 levels and downregulating Bax levels.

## 5. Conclusions

DMY within our range of observed concentrations significantly ameliorated cisplatin-induced nephrotoxicity via attenuating cisplatin-induced oxidative stress, protected against cisplatin-induced apoptotic cell death, and alleviated inflammatory reactions in mouse kidney tissues. Although we studied several underlying mechanisms involved in this process, further studies are required to explore the other mechanism(s) of the responses evoked by DMY.

## Figures and Tables

**Table 1:** Primers for Q-PCR.

Primers	Forward: 5′-3′	Reverse: 5′-3′	Product length (bp)
IL-6	AAGGAGTGGCTAAGGACCAA	GTTTGCCGAGTAGATCTCAAA	**89**
IL-1*β*	TTCCTTGTGCAAGTGTCTGAAG	CACTGTCAAAAGGTGGCATTT	**76**
TNF-*α*	TGACGTGGAACTGGCAGAAGA	TGGGCCATAGAACTGATGAGAG	**204**
TGF-*β*	CGTGGAAATCAACGGG	CAGAAGTTGGCATGGT	**267**
MCP-1	GCTGACCCCAAGAAGGAATG	GAAGACCTTAGGGCAGATGCA	**110**
*β*-Actin	CATGTACGTTGCTATCCAGGC	CTCCTTAATGTCACGCACGAT	**250**

**Table 2:** Effect of Dihydromyricetin on oxidative stress in different experimental groups.

Groups	MDA (*μ*M/g tissue)	SOD (U/mg protein)	CAT (U/mg protein)
Normal	4.21 ± 1.60	21.27 ± 1.99	9.97 ± 1.47
DMY 500	4.15 ± 1.07	23.78 ± 3.82	9.55 ± 2.15
Cis	24.57 ± 4.73^*∗∗*^	8.80 ± 2.48^*∗∗*^	3.29 ± 0.97^*∗∗*^
Cis+DMY 500	15.20 ± 3.77^#^	16.48 ± 1.59^##^	6.03 ± 1.15^#^

Cis: cisplatin; DMY 500: DMY 500 mg/kg; Cis+DMY 500: cisplatin+DMY 500 mg/kg. Data is expressed as mean ± SD of 4 mice per group. MDA: malondialdehyde, SOD: superoxide dismutase, CAT: catalase activity. ^*∗∗*^ *P* < 0.01 versus normal group. ^##^ *P* < 0.01 versus cisplatin treatment group. ^#^ *P* < 0.05 versus cisplatin treatment group.
